# Association between functional polymorphisms in the promoter of the miR-143/145 cluster and risk of intracranial aneurysm

**DOI:** 10.1038/srep43633

**Published:** 2017-03-08

**Authors:** Xiutian Sima, Hong Sun, Peizhi Zhou, Chao You, Bowen Cai

**Affiliations:** 1 Department of Neurosurgery, West China Hospital of Sichuan University, Chengdu, Sichuan, 610041, P. R. China

## Abstract

MicroRNAs (miRs)-143/145 are involved in various biological processes related to aneurysm formation and are downregulated in patients with intracranial aneurysm (IA). We aimed to determine whether two functional polymorphisms (i.e. rs4705342 and rs4705343) in the promoter of miR-143/145 are related to IA risk. A case-control study was undertaken to examine the association of rs4705342 and rs4705343 with IA risk, including 565 patients with IA and 622 age- and gender-matched controls. rs4705342 was analysed by TaqMan Assay, and rs4705343 was genotyped using polymerase chain reaction-restriction fragment length polymorphism. miR-143/145 expression was quantified using RT-PCR. rs4705342 was associated with a significantly lower risk of IA, with adjusted ORs of 0.74 (95% CI: 0.58–0.95) for TC genotype carriers and 0.74 (95% CI: 0.59–0.94) for TC/CC genotypes carriers. Individuals carrying the rs4705342 C allele had a reduced risk of IA (adjusted OR = 0.82; 95% CI: 0.68–0.98). Haplotype of the two loci of rs4705342 and rs4705343 showed that the CT haplotype carried a lower IA risk and higher miR-143 level. Moreover, the rs4705342 CC/CT genotypes were associated with higher miR-143 levels. Thus, the rs4705342C-rs4705343T haplotype in the promoter of miR-143/145 cluster may be related to IA development.

Subarachnoid haemorrhage (SAH) is a serious condition that leads to death in approximately 30% patients in the initial weeks after bleeding onset and severe physical or mental impairment in the rest^1^,^2^. The major cause of SAH is rupture of intracranial aneurysms (IAs), which accounts for 85% of spontaneous SAH cases^3^.

Risk factors associated with IA include advanced age, female gender, hypertension, cigarette smoking and excessive alcohol intake^4^,^5^,^6^. In addition to these environmental risk factors, a genetic component has been recognized to play a role in IA development. For example, the first-degree relatives of patients with aneurysms are at a higher risk of IA development^7^. Asymptomatic familial aneurysms are more likely to rupture than non-familial ones^8^. We previously reported that the rs4938723 CC genotype in the promoter of pri-miR-34b/c and the rs13293512 CT genotype in the promoter of let-7 were associated with the risk of IA^9^,^10^.

MicroRNAs (miRNAs), which are non-coding RNAs approximately 19–23 nucleotides long, have been identified as key regulators in a host of biological processes^11^. Genome-wide miRNA screening analysis showed that more than 100 miRNAs were differentially expressed in human IA tissues^12^, among which miR-143 and miR-145 were reported to be downregulated and involved in the pathophysiology of aneurysms, including processes such as inflammation, dysregulation of the extracellular matrix, smooth muscle cell (SMC) proliferation, programmed cell death and response to oxidative stress^12^,^13^,^14^. Thus, miR-143 and miR-145 may play pivotal roles in the occurrence of IA.

Recent evidence has shown that genetic variants in the promoters of miRNAs may affect miRNA expression, contributing to individuals’ susceptibility to diseases as well as disease outcomes^9^,^15^,^16^. In 2013, several single nucleotide polymorphisms (SNPs) were discovered in the promoter of the miR-143/145 cluster^17^. Subsequent investigations showed that of them, rs4705342 and rs4705343 were functional^18^,^19^. The rs4705342 risk-associated T allele, located 510 bp upstream from the transcription start site, increases protein-binding affinity and decreases promoter activity^18^. On the other hand, the rs4705343 risk-associated C allele, located 400 bp upstream from the transcription start site, reduces luciferase activity^19^. These two SNPs were reported to be associated with the risk of essential hypertension, prostate cancer and cervical squamous cell carcinoma^18^,^19^,^20^. No study, to date, has investigated the association between these two polymorphisms and IA risk. The present study was designed to evaluate the correlation between rs4705342 and rs4705343 and IA risk in a Chinese population. The effects of these SNPs on the plasma levels of miR-143 and miR-145 were also examined.

## Materials and Methods

### Study population

Quanto software was used to estimate the required sample size. To ensure a statistical power of over 80%, the sample size must exceed 550 if the odds ratio (OR) is set at 1.40 under a dominant model. We examined a total of 1187 subjects, including 565 IA patients and 622 age- and gender-matched controls. All examined individuals were unrelated Chinese Han. Patients were recruited consecutively from the Department of Neurosurgery of the West China Hospital of Sichuan University between January 2008 and July 2016. The diagnosis of IA was established using digital subtraction angiography. Patients were excluded if they had hypertension, head trauma, intracranial atherosclerosis and/or other nervous system diseases. Control subjects were healthy volunteers living in Sichuan province or the surrounding area during the study period. The exclusion criteria for the controls were hypertension and some common nervous system diseases and disorders, such as stroke, traumatic brain injury, Alzheimer disease and Parkinson disease.

### Ethics statement

The study protocol was approved by the Institutional Review Board of West China Hospital of Sichuan University. Informed consent was obtained from all participants prior to testing. If patients were unable to write, the consent form was signed by their relatives. All experiments were performed in accordance with institutional guidelines and were approved by the West China Hospital of Sichuan University.

### DNA isolation

Two to three millilitres of peripheral blood samples were collected from each subject into ethylene diamine tetraacetic acid-containing vacuumed tubes. Whole blood was centrifuged at 1600 *g* for 10 min at 4 °C, and the plasma was separated and frozen at −80 °C until analysis. Genomic DNA was extracted from peripheral blood leukocytes according to the manufacturer’s instructions (Bioteke, Beijing, China) and stored at −20 °C.

### Analysis of polymorphism

Genotyping analysis for rs4705342 was performed using the TaqMan Assay on an ABI 7900HT sequence detection system (Applied Biosystems, CA, USA)^18^, while that for rs4705343 was performed using polymerase chain reaction-restriction fragment length polymorphism^19^. The primers, probes and enzymes used were processed as described previously^18^,^19^. For internal quality control, about 5% samples were selected for Sanger sequencing, and the concordance rates among these quality control samples were found to be 100% between the assays.

### Quantitative RT-PCR

Total RNA was isolated from the plasma samples of 62 patients with IA and 62 controls using a QIAamp circulating nucleic acid kit according to the manufacturer’s instructions (Qiagen, Hilden, Germany). To determine the expression of miR-143 and miR-145, we used a QuantiFast SYBR Green PCR Kit (Qiagen, Hilden, Germany). Briefly, after reverse transcription of total RNA, cDNA was generated and subjected to quantitative RT-PCR. The PCR conditions were as follows: initial denaturation (95 °C for 4 min) followed by 40 PCR cycles (95 °C for 10 s and 60 °C for 30 s). The PCR primers for miR-143 and miR-145 were bought from Ribobio Corp. (Guangzhou, China). U6 was used as an internal control, and nuclease-free water was used as a negative control. The threshold cycle (Ct) is the cycle number at which the fluorescent signal passes a fixed threshold. The relative expression of miR-143 and miR-145 was measured using the 2^−ΔCt^ method^21^.

### Statistical analyses

The χ^2^ test was used to examine departure from the Hardy–Weinberg equilibrium (HWE) among the controls. To evaluate the relative risk of IA in relation to rs4705342 and rs4705343, ORs and 95% confidence intervals (CIs) were computed using unconditional logistic regression, after adjusting for age, gender and familial aneurysms. For genotype or allele comparison, the common homozygote genotype or allele was set as a reference, respectively. Linkage disequilibrium (LD) and haplotype analyses were performed using SHEsis software (http://analysis.bio-x.cn/myAnalysis.php)^22^, for which the most common haplotype was set as a reference. ORs and 95% CIs for haplotype analysis were calculated based on the frequencies obtained from the SHEsis software. Plasma levels of miR-143 and miR-145 were compared between cases and controls using the Mann-Whitney *U* test. All statistical analyses were performed using SPSS software, version 19.0 (SPSS Inc., Chicago, IL). All statistical tests were two sided with a significance level of 0.05.

## Results

### Characteristics of the study population

The characteristics of the 565 cases and 622 controls are summarized in Table 1. Demographic information, including age and gender, was comparable between the groups (*P* = 0.30 and 0.38, respectively). Most IAs were single intracranial aneurysms (86.2%), ruptured aneurysms (88.0%) and non-familial aneurysms (90.3%).

### Association of rs4705342 and rs4705343 with IA risk

Among the controls, genotype distributions for rs4705342 and rs4705343 followed the HWE (*P* = 0.48 and 0.09, respectively). The rs4705342 was significantly associated with a decreased IA risk, with adjusted ORs of 0.74 (95% CI: 0.58–0.95, *P* = 0.017) for TC genotype carriers and 0.74 (95% CI: 0.59–0.94, *P* = 0.013) for TC/CC genotypes carriers, when compared with the more common TT genotype carriers. Moreover, individuals carrying the rs4705342 C allele had a reduced risk of IA relative to those carrying the T allele (adjusted OR = 0.82; 95% CI: 0.68–0.98, *P* = 0.03). However, no clear association was observed between rs4705343 and IA risk (Table 2).

For haplotype analysis of rs4705342 and rs4705343, four haplotypes were reconstructed from genotypic data by using SHEsis software. Strong LD was found between the two polymorphic sites (D′ = 0.74, r^2^ = 0.51). The CT haplotype containing the rs4705342 C allele was associated with a lower IA risk compared with the most frequent TT haplotype (OR = 0.18, 95% CI: 0.11–0.30, *P* < 0.001) (Table 3).

### Association of rs4705342 and rs4705343 with plasma levels of the miR-143/145 cluster

**Figure 1:**
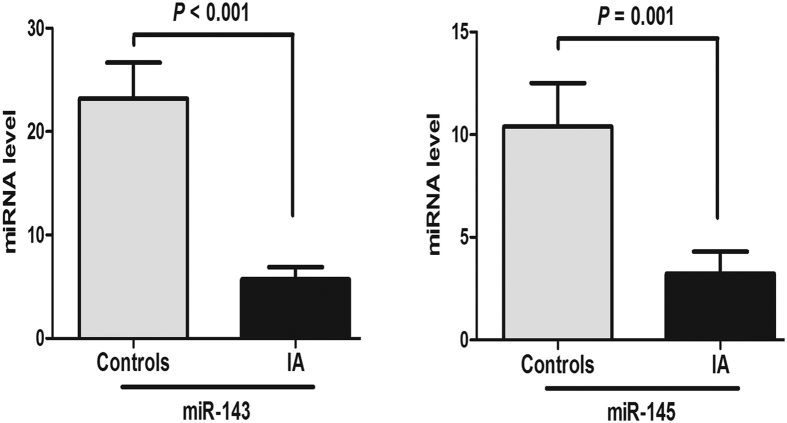
Relative plasma levels of miR-143 and miR-145 in controls and intracranial aneurysm (IA) patients (n = 62). Data are expressed as mean ± SEM.

**Figure 2:**
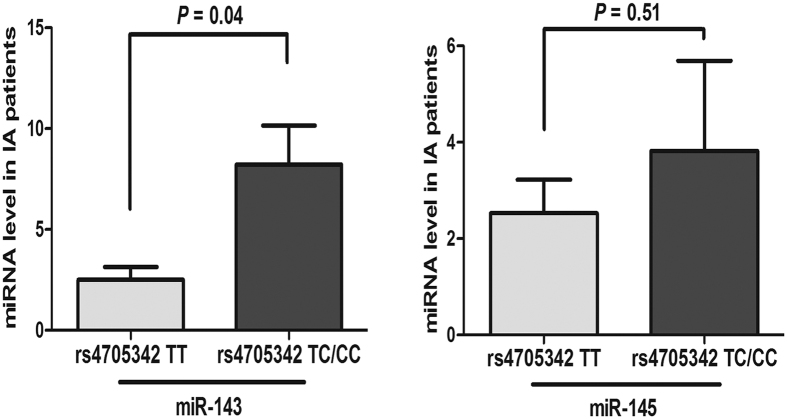
Association between rs4705342 and plasma levels of miR-143 and miR-145 in intracranial aneurysm (IA) patients. Data are expressed as mean ± SEM.

**Figure 3:**
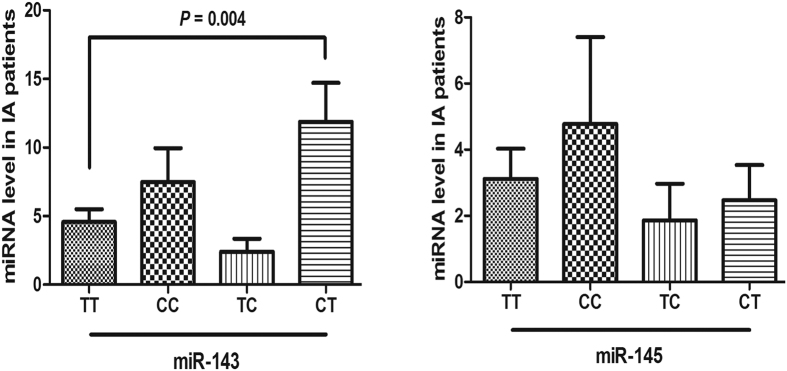
Association between rs4705342-rs4705343 haplotypes and plasma levels of miR-143 and miR-145 in intracranial aneurysm (IA) patients. Data are expressed as mean ± SEM.

To explore the effect of rs4705342 and rs4705343 on the expression of the miR-143/145 cluster, we examined the plasma levels of miR-143 and miR-145 in patients with IA and controls. The results of qPCR showed that the expression of miR-143 and miR-145 in patients with IA was lower than that in the controls (*P* < 0.001 and *P* = 0.001, respectively) (Fig. 1). The patients were then classified into two groups based on genotype: TT and TC/CC. Compared to rs4705342 TT genotype carriers, CC/CT genotypes carriers had higher levels of miR-143 (*P* = 0.04) but not miR-145 (Fig. 2). After comparing the levels of miR-143 and miR-145 among groups classified by haplotypes, we found that CT haplotpye exhibited a higher level of miR-143 than TT haplotype (*P* = 0.004) (Fig. 3). rs4705343 had no effect on the levels of either miR-143 or 145.

## Discussion

In the present study, we examined the effect of two functional SNPs in the promoter of the miR-143/145 cluster on the development of IA. We found that the risk of IA was lower in individuals with the rs4705342 TC and TC/CC genotypes than those with the TT genotype. Individuals with the rs4705342 C allele showed a 0.82-fold reduced risk of IA. Haplotype analysis showed that those with the CT haplotype had a lower risk of IA and higher miR-143 level. Notably, rs4705342 CC/CT genotypes carriers had a higher plasma level of miR-143 than rs4705342 TT genotype carriers. Collectively, the results indicate that the rs4705342C-rs4705343T haplotype in the promoter of the miR-143/145 cluster may be related to the aetiology of IA.

It has been identified that atherosclerotic like changes are observed in the formation of IA, such as myointimal or neointimal hyperplasia, SMC proliferation, vascular wall remodeling, lipid accumulation and foam cell formation^23^,^24^,^25^,^26^. Many miRNAs have been found to play crucial roles in vascular physiology. A notable example is the miR-143/145 cluster, which is strongly expressed in SMCs, acting as an anti-atherosclerotic in a paracrine manner^27^,^28^,^29^,^30^,^31^. miR-143 and miR-145 have been shown to be protective during vascular remodelling and carotid plaque progression and stabilization^32^,^33^,^34^. In miR-143/145-null mice, vascular SMCs are locked in the synthetic state that favor neointimal lesion development^28^. Restoration of miR-143/145 can promote reduction and stabilization of atherosclerotic plaques and reduce neointimal hyperplasia^35^,^36^. Moreover, miR-145 may be a vascular SMC phenotypic marker and modulator that can control vascular neointimal lesion formation through its target gene Kruppel-like factor 5 and its downstream signalling molecule myocardin^37^,^38^. In addition to its key role in atherosclerosis, the miR-143/145 cluster has been shown to be downregulated in IA, triggering a series of biological processes related to aneurysm formation^12^,^13^,^14^. However, Sala *et al*. reported that miR-143/145 deficiency attenuated the progression of atherosclerosis^39^, indicating that this cluster may have both positive and negative roles in the formation of atherosclerosis.

A previous genome-wide association study of IA identified a risk locus at 5q23.2^40^. miR-143 and miR-145 are located on chromosome 5 in the human genome. We hypothesized that two functional SNPs (i.e. rs4705342 and rs4705343) in the promoter of the miR-143/145 cluster may be related to the risk of IA, and our results confirmed the hypothesis. We found that rs4705342 TC/CC genotypes were associated with reduced IA susceptibility. Our findings were in agreement with those of a previous study that showed a reduced risk of prostate cancer^18^ as well as essential hypertension^20^ in individuals with the rs4705342 C allele. With regard to rs4705343, we did not find any association between this polymorphism and IA risk. However, Liang *et al*. found that individuals with the rs4705343 TC genotype had a 1.37-fold higher risk of cervical squamous cell carcinoma^19^, indicating that this SNP plays different roles in different diseases. Additionally, we found that individuals with the rs4705342C-rs4705343T haplotype were less likely to develop IA. This result may be biologically reasonable because genome-wide linkage and haplotype-association studies on IA found evidence of linkage on chromosome 5q22-31^41^.

To explore a possible reason for why individuals with the rs4705342 CC/CT genotypes have a low IA risk, we measured the plasma levels of miR-143/145 and performed a genotype-phenotype analysis. We found that both miR-143 and miR-145 were down-regulated in patients with IA, proving that the miR-143/145 cluster has protective effects against IA. Importantly, we found that the rs4705342 CC/CT genotypes were associated with high miR-143 levels. We hypothesized that this SNP may affect the functioning of SMCs and the development of atherosclerotic changes, and eventually influence individuals’ susceptibility to IA. Further studies are necessary to confirm this hypothesis.

In the present study, we proved that rs4705342 TC/CC genotypes are protective against IA. However, these data were obtained from the Chinese Han population. Therefore, the findings cannot be directly extended to other ethnicities and must be confirmed in various ethnic groups. As is known, smoking and alcohol consumption are risk factors for IA. Gene-environment interaction analysis, however, could not be evaluated in this study because some data were missing or subjective. The control subjects in this study did not undergo magnetic resonance angiography or any other screening, and therefore we cannot exclude the possibility of selection bias. Lastly, the exact mechanism by which rs4705342 is involved in IA is not understood in detail. Further investigations are important for this purpose.

In conclusion, we found for the first time that the rs4705342 TC and TC/CC genotypes in the promoter of the miR-143/145 cluster are related to a lower risk of IA. It would be of great interest to further evaluate the possible mechanism by which rs4705342 affects IA risk, as this will eventually help gain deeper insights into SNPs relevant to the development of IA and other human diseases.

## Additional Information

**How to cite this article:** Sima, X. *et al*. Association between functional polymorphisms in the promoter of the miR-143/145 cluster and risk of intracranial aneurysm. *Sci. Rep.* **7**, 43633; doi: 10.1038/srep43633 (2017).

**Publisher's note:** Springer Nature remains neutral with regard to jurisdictional claims in published maps and institutional affiliations.

## Figures and Tables

**Table 1:** Demographics of controls and patients with IA.

	Controls, n = 622	Patients with IA, n = 565	*P* value
Age (years, mean ± SD)	51.0 ± 9.0	51.7 ± 11.8	0.30
Gender			
Male	231 (37.1)	224 (39.6)	0.38
Female	391 (62.9)	341 (60.4)	
Multiple aneurysm			
Yes		78 (13.8)	
No		487 (86.2)	
Rupture of aneurysm			
Yes		497 (88.0)	
No		68 (12.0)	
Familial aneurysm			
Yes		55 (9.7)	
No		510 (90.3)	

IA, intracranial aneurysm; SD, standard deviation.

**Table 2:** Genotype frequencies of rs4705342 and rs4705343 in patients with IA and controls.

Polymorphisms	Controls, n = 622 (%)	Patients, n = 565 (%)	Adjusted OR (95% CI)^†^	*P* value^†^
rs4705342				
TT	277 (44.5)	289 (51.1)	1.00	
TC	282 (45.3)	227 (40.2)	0.74 (0.58–0.95)	0.017
CC	63 (10.1)	49 (8.7)	0.75 (0.49–1.14)	0.17
TC/CC	345 (55.5)	276 (48.9)	0.74 (0.59–0.94)	0.013
T allele	836 (67.2)	805 (71.2)	1.00	
C allele	408 (32.8)	325 (28.8)	0.82 (0.68–0.98)	0.03
rs4705343				
TT	291 (46.8)	244 (43.2)	1.00	
TC	282 (45.3)	264 (46.7)	1.10 (0.86–1.41)	0.43
CC	49 (7.9)	57 (10.1)	1.38 (0.90–2.11)	0.15
TC/CC	331 (53.2)	321 (56.8)	1.15 (0.91–1.45)	0.25
T allele	864 (69.5)	752 (66.5)	1.00	
C allele	380 (30.6)	378 (33.5)	1.14 (0.96–1.37)	0.14

IA, intracranial aneurysm; OR: odds ratio; CI: confidence interval; ^†^Adjusted by age, gender, and familial aneurysm.

**Table 3:** Haplotype analysis of rs4705342 and rs4705343 with IA risk.

Haplotypes		Controls (%)	IA (%)	OR (95% CI)	*P* value
rs4705342	rs4705343				
T	T	751 (60.4)	732 (64.8)	1.00	
C	C	296 (23.8)	305 (27.0)	1.06 (0.88–1.28)	0.57
T	C	85 (6.8)	73 (6.5)	0.88 (0.63–1.22)	0.45
C	T	112 (9.0)	20 (1.8)	0.18 (0.11–0.30)	<0.001

IA, intracranial aneurysm; OR, odds ratio; CI, confidence interval.
